# Chlorhexidine 0.12% Mouthwash With or Without Bioflavonoids as an Adjunct to Non‐Surgical Periodontal Therapy: A Randomized Clinical Trial

**DOI:** 10.1111/jre.13423

**Published:** 2025-05-24

**Authors:** Margherita Giorgia Liguori, Franz Josef Strauss, Francesco Tarallo, Davide Simeone, Alessandro Crea, Enrico Marchetti

**Affiliations:** ^1^ Department of Life, Health and Environmental Sciences University of L'aquila L'Aquila Italy; ^2^ Center for Dental Medicine University of Zurich Zurich Switzerland; ^3^ Universidad Autonoma de Chile Talca Chile; ^4^ Private Practice Viterbo Italy

**Keywords:** bioflavonoids, chlorhexidine, mouthwash, non‐surgical periodontal therapy, patient‐reported outcomes, periodontitis

## Abstract

This randomized clinical trial evaluated the adjunctive use of bioflavonoids in a 0.12% chlorhexidine mouthwash during non‐surgical periodontal therapy. The addition of bioflavonoids resulted in clinical and patient‐reported outcomes comparable to chlorhexidine alone, with both groups showing improvement over time and good overall treatment tolerance.

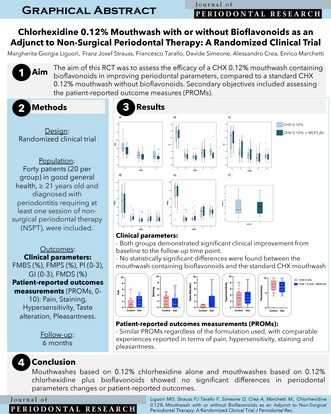

**Summary**

- The mouthwash containing 0.12% chlorhexidine with the adjunct of bioflavonoids did not result in improved clinical or patient‐reported outcomes (PROs) compared to the 0.12% chlorhexidine mouthwash alone.

## 1 Introduction

Non‐surgical periodontal therapy (NSPT) is a standard treatment for all periodontal patients but presents limitations, including incomplete removal of bacterial deposits from deep pockets, intrabony defects, and furcation areas [1]. In complex cases, adjunctive chemical agents may be used to enhance plaque control and reduce gingival inflammation [1, 2]. Chlorhexidine digluconate (CHX) has consistently shown to be an effective adjunct to NSPT. However, its long‐term use is limited by side effects [3].

Bioflavonoids are naturally occurring compounds with antioxidant, anti‐inflammatory, and antimicrobial properties [4, 5]. Integrating bioflavonoids could enhance the effects of CHX and may help mitigate CHX‐related side effects, improving overall satisfaction. The aim of this randomized controlled trial was to compare the clinical and patient‐reported outcomes of NSPT using a 0.12% CHX mouthwash containing bioflavonoids versus a standard 0.12% CHX mouthwash.

## 2 Methods

This randomized clinical trial was conducted in accordance with the Declaration of Helsinki and approved by the University's Internal Review Board (PROT45081). It was registered at ClinicalTrials.gov (NCT06341439) and reported according to CONSORT guidelines. Patients were enrolled between March and August 2024, regardless of periodontitis stage and grade, and randomly assigned (1:1) to two groups:

- *TEST*–mouthwash containing 0.12% CHX and bioflavonoids (*Curaprox PerioPlus + Protect*, Curaden AG, Switzerland);
- *CONTROL*–mouthwash containing 0.12% CHX, with the same formulation as the test mouthwash, without bioflavonoids.

Eligible participants were ASA I‐II, over 21 years old, non‐ or light smokers (< 5 cig/day), > 10 natural teeth, and at least two sites with probing depth ≥ 5 mm. Exclusion criteria included severe systemic diseases, head and neck radiotherapy, uncontrolled diabetes or hypertension, allergy to CHX, medications affecting oral tissues, and pregnancy or breastfeeding.

Following NSPT (T_1_), patients were randomly allocated and instructed to rinse with the assigned mouthwash (300 mL) twice daily for 60 s over 14 days. Follow‐up assessments were conducted at 14 days (T_2_), 2 months (T_3_), and 6 months (T_4_). The following parameters were recorded: probing depth (PD), full mouth bleeding score (FMBS), full mouth plaque score (FMPS), full mouth discoloration score (FMDS), gingival index (GI), and plaque index (PI) [6, 7]. Patient‐reported outcome measures (PROMs) were evaluated at T_2_ for pain, staining, taste alteration, hypersensitivity, and pleasantness (VAS; 0–10). All follow‐up visits were conducted by the same experienced periodontist who was blinded to the group assignment.

A sample size calculation was conducted prior to the start of the study. A total of 40 patients were planned based on local recruitment feasibility and the rule‐of‐thumb recommendation [8]. FMBS was the primary outcome, and this sample also aligns with an effect size of 1.0, 80% power, 5% alpha, and 15% dropout. Statistical analysis included descriptive statistics and the Shapiro–Wilk test. When normality was met, group‐time interactions were analyzed using a two‐way ANOVA‐type statistic. When assumptions were violated, the Brunner‐Langer non‐parametric model for longitudinal data was applied. Pairwise comparisons were conducted using Wilcoxon and Mann–Whitney *U* tests with Bonferroni correction. Significance was set at *p* < 0.05.

## 3 Results

A total of 43 patients were screened for eligibility. Forty completed the study and were included in the analysis (20 per group). At baseline, both groups were comparable in age (*p* = 0.250), sex distribution (*p* = 0.204), and smoking status (*p* = 0.288). Stage III was most common (75% in both groups), followed by Stage II (10%–15%) and Stage IV (10%–15%). Grade C predominated in both groups, with 70% in the control and 60% in the test group (Table 1).

**TABLE 1:** Mean values of clinical parameters between study groups at each time point.

Variable	Time point	CHX 0.12% (*n* = 20)	CHX 0.12% + BIOFLAV (*n* = 20)	*p* between groups
FMBS (%)	Baseline	52.8 ± 27.4	52.8 ± 27.4	0.387 (NS)
	2 weeks post‐NSPT	10.7 ± 8.1	9.5 ± 7.2	
	2 months post‐NSPT	12.1 ± 7.7	11.4 ± 8.8	
	6 months post‐NSPT	11.6 ± 7.3	12.1 ± 7.6	
	Δ (6 months − Baseline)	41.15 ± 27.71	37.86 ± 24.84	
	*p* intra group	**< 0.001** *	**< 0.001** *	
FMPS (%)	Baseline	75.0 ± 20.9	76.2 ± 23.8	0.387 (NS)
	2 weeks post‐NSPT	14.6 ± 9.6	12.6 ± 7.2	
	2 months post‐NSPT	29.1 ± 11.3	27.3 ± 11.6	
	6 months post‐NSPT	28.5 ± 7.1	28.7 ± 8.3	
	Δ (6 months − Baseline)	46.50 ± 22.26	47.35 ± 25.12	
	*p* intra group	**< 0.001** *	**< 0.001** *	
GI (0‐3)	Baseline	2.05 ± 0.67	1.91 ± 0.71	0.512 (NS)
	2 weeks post‐NSPT	0.77 ± 0.52	0.70 ± 0.34	
	2 months post‐NSPT	0.65 ± 0.43	0.57 ± 0.38	
	6 months post‐NSPT	0.59 ± 0.27	0.67 ± 0.34	
	Δ (6 months − Baseline)	1.48 ± 0.67	1.17 ± 0.71	
	*p* intra group	**< 0.001** *	**< 0.001** *	
PI (0‐3)	Baseline	2.28 ± 0.46	2.27 ± 0.57	0.313 (NS)
	2 weeks post‐NSPT	0.56 ± 0.25	0.63 ± 0.26	
	2 months post‐NSPT	0.81 ± 0.24	0.92 ± 0.40	
	6 months post‐NSPT	0.77 ± 0.37	0.97 ± 0.36	
	Δ (6 months − Baseline)	1.51 ± 0.53	1.30 ± 0.60	
	*p* intra group	**< 0.001** *	**< 0.001** *	

*Note:* Overall *p*‐values were obtained using the Brunner–Langer model (ANOVA‐type statistic). Pairwise comparisons were assessed with the Wilcoxon signed‐rank test and Bonferroni correction.

Abbreviation: NS = not significant.

* *p* < 0.001 versus baseline.

Both groups showed statistically significant improvements in all clinical parameters from baseline to the 6‐month follow‐up (*p* < 0.01). FMBS decreased from approximately 52% to 12% in both groups, and FMPS from ~75% to ~28%. PD, GI, and PI also declined over time (Tables 1 and 2). At day 14, FMDS were similar between groups (*p* > 0.05). Mild dental staining was observed in more than 40% of patients, but was well tolerated and did not result in study withdrawal. PROMs revealed no statistically significant differences between groups (Table S1). No treatment‐related adverse effects were reported.

**TABLE 2:** Shallow, moderate, and deep pockets at baseline and following non‐surgical periodontal therapy (NSPT).

	CHX 0.12% (*n* = 20)			CHX 0.12% + BIOFLAV (*n* = 20)		
Sites	Baseline	2 months	6 months	Baseline	2 months	6 months
PPD ≤ 3 mm (%)	1337 (40)	2320 (70)	2257 (68)	1618 (51)	2351 (75)	2406 (76)
PPD 4–5 mm (%)	1285 (38)	798 (24)	860 (26)	1156 (36)	655 (21)	678 (21)
PPD ≥ 6 mm (%)	744 (22)	188 (6)	193 (6)	424 (13)	132 (4)	109 (3)

## 4 Discussion

This trial evaluated the adjunctive effect of a 0.12% chlorhexidine (CHX) mouthwash containing bioflavonoids. The use of CHX is supported by the S3‐level clinical practice guideline as a short‐term adjunct in selected cases [1, 9]. Although bioflavonoids exhibit promising in vitro antimicrobial and anti‐inflammatory effects, no additional clinical or patient‐reported benefits were observed in this setting [5]. Interestingly, a slight but statistically significant worsening of FMBS at T_2_ was observed in both groups, possibly reflecting a rebound in gingival inflammation following cessation of CHX. Some limitations of this study should be acknowledged. The small sample size may have reduced the ability to detect subtle differences; the absence of a negative control group limits interpretation of the adjunctive effect. In addition, as this was a single‐center trial, external validity may be limited.

In conclusion, both groups showed improvements, but the adjunctive use of bioflavonoids in a 0.12% CHX mouthwash did not enhance clinical or patient‐reported outcomes. Further trials are needed to explore the potential benefit of bioflavonoids.

## Author Contributions

All authors have made substantial contributions to the conception and design of the study. M.G.L. conceived the study design. F.T. and A.C. were involved in data collection and analysis. F.J.S. contributed to data analysis and interpretation. M.G.L. and D.S. drafted the manuscript, while E.M. critically revised it and provided final approval of the version to be published.

## Ethics Statement

This clinical trial was approved by the IRB of University of L'Aquila (Approval Number: PROT45081) and registered at clinicaltrials.gov under the registration number NCT06341439.

## Supporting information


**Table S1:** Patient‐reported Outcome Measures (PROMs).
