# Supplementary material for: Chlorhexidine 0.12% Mouthwash With or Without Bioflavonoids as an Adjunct to Non‐Surgical Periodontal Therapy: A Randomized Clinical Trial
Source: J Periodontal Res. 2025 May 24;60(9):937–9. doi: 10.1111/jre.13423 (PMC12517244; doi:10.1111/jre.13423)
Supplement: Supplementary file 1 — Table S1: Patient‐reported Outcome Measures (PROMs). [file JRE-60-937-s001.docx]

**Supplemental Table 1.** Patient-reported Outcome Measures (PROMs)

|  | | **GROUP** | | |  |
| --- | --- | --- | --- | --- | --- |
|  |  | **Total** | **Control Group** | **Test group** | ***p*** |
| **PAIN** | **N** | 40 | 20 | 20 | 0.121 |
|  | **Mean** | 1.7 | 1.3 | 2.1 |  |
|  | **Standard Deviation** | 1.9 | 1.9 | 1.9 |  |
|  | **Median** | 1.0 | .0 | 2.0 |  |
| **STAINING** | **N** | 40 | 20 | 20 | 0.779 |
|  | **Mean** | 1.9 | 2.1 | 1.7 |  |
|  | **Standard Deviation** | 2.3 | 2.6 | 2.0 |  |
|  | **Median** | 1.0 | 1.0 | 1.0 |  |
| **TASTE ALTERATION** | **N** | 40 | 20 | 20 | 0.314 |
|  | **Mean** | 1.1 | .4 | 1.9 |  |
|  | **Standard Deviation** | 2.1 | .5 | 2.7 |  |
|  | **Median** | .0 | .0 | .0 |  |
| **HYPERSENSITIVITY** | **N** | 40 | 20 | 20 | 0.925 |
|  | **Mean** | 3.3 | 3.2 | 3.4 |  |
|  | **Standard Deviation** | 2.7 | 2.7 | 2.8 |  |
|  | **Median** | 3.0 | 3.0 | 2.5 |  |
| **PLEASANTNESS** | **N** | 40 | 20 | 20 | 0.429 |
|  | **Mean** | 7.3 | 7.5 | 7.0 |  |
|  | **Standard Deviation** | 1.9 | 1.7 | 2.1 |  |
|  | **Median** | 7.0 | 8.0 | 7.0 |  |

p-values from Mann-Whitney´s test
